# Red-shifted click beetle luciferase mutant expands the multicolor bioluminescent palette for deep tissue imaging

**DOI:** 10.1016/j.isci.2020.101986

**Published:** 2020-12-26

**Authors:** Giorgia Zambito, Mary P. Hall, Monika G. Wood, Natasa Gaspar, Yanto Ridwan, Fabio F. Stellari, Ce Shi, Thomas A. Kirkland, Lance P. Encell, Clemens Löwik, Laura Mezzanotte

**Affiliations:** 1 Erasmus Medical Center, Radiology and Nuclear Medicine, Rotterdam, 3015 CE, the Netherlands; 2 Erasmus Medical Center, Molecular Genetics, Rotterdam, 3015 CE, the Netherlands; 3 Medres Medical Research GmBH, Cologne 50931, Germany; 4 Promega Corporation, Madison, WI 53711, USA; 5 Percuros B.V., Leiden, 1333 CL, the Netherlands; 6 Chiesi Farmaceutici, Parma, 43122, Italy; 7 Promega Biosciences Incorporated, San Luis Obispo, CA 93401, USA; 8 CHUV Department of Oncology, University of Lausanne, 461011 Lausanne, Switzerland

**Keywords:** Optical Imaging, Biological Services, Biophysics

## Abstract

For *in vivo* multicolor bioluminescence applications, red and near-infrared signals are desirable over shorter wavelength signals because they are not as susceptible to light attenuation by blood and tissue. Herein, we describe the development of a new click beetle luciferase mutant, CBG2, with a red-shifted color emission. When paired with NH_2_-NpLH2 luciferin, CBG2 (λ = 660 nm) and CBR2 (λ = 730 nm) luciferases can be used for simultaneous dual-color bioluminescence imaging in deep tissue. Using a spectral unmixing algorithm tool it is possible to distinguish each spectral contribution. Ultimately, this enzyme pair can expand the near-infrared bioluminescent toolbox to enable rapid visualization of multiple biological processes in deep tissue using a single substrate.

## Highlights

• CBG2 is a new click beetle mutant derived from CBG99 luciferase
• It has a far red-shifted bioluminescent emission with NH_2_-NpLH2 luciferin (λ = 660 nm)
• CBG2 with CBR2 luciferase and NH_2_-NpLH2 allows fast acquisition of data in deep tissue

## Graphical Abstract

**Figure:**
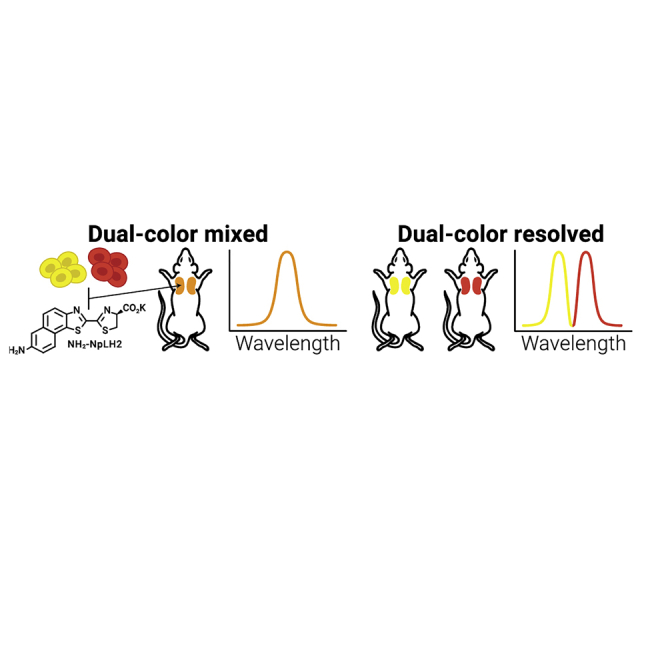

## Introduction

Bioluminescence imaging (BLI) has become a highly adopted technique for preclinical and non-invasive study of biological events *in vivo* (Kaskova et al., 2016; Mezzanotte et al., 2017). The production of bioluminescence depends on luciferase-enzyme-catalyzed oxidation of a luciferin substrate (Wilson and Hastings, 1998). The use of luciferases emitting photons in the “bio-optical window” (λ = 600 nm–800 nm) is highly recommended to limit light absorption by tissue components *in vivo* (Jathoul et al., 2014; Smith et al., 2009). Thus, red-shifted luciferase mutants improve the sensitivity of BLI and allow tracking of single cells over time in deep tissue (Branchini et al., 2010; Iwano et al., 2018). However, it is still challenging to visualize multiple biological processes over time in deep tissue because current BLI offerings are limited. In many of the systems currently used, sequential administration of multiple substrates is required, making interpretation of data challenging (Maguire et al., 2013; Taylor et al., 2018). Previously, we attempted dual-color BLI using green click beetle (CBG99) and red firefly (PpyRe8) luciferases with D-LH2. However, the signal for CBG99 was attenuated in deep tissue, resulting in acquisition of predominantly the red contribution (Mezzanotte et al., 2011).

An ideal approach for deep tissue multicolor BLI would be to utilize a single substrate with two luciferases emitting spectrally separated signals in the near-IR bio-optical window. Notably, the recent development of infra-luciferin (iLH2) proved to shift the FLuc mutants to the far-red and near-infrared region of spectrum (FLuc_green ∼680 nm and FLuc_red ∼720 nm) (Jathoul et al., 2014; Branchini et al., 2007). Stowe et al. demonstrated that engrafted red-CAR T cells can expand and reach the green-Raji B lymphoma when iLH2 is used *in vivo* (Stowe et al., 2019). Green and red signals were acquired with a sensitive CCD camera and quantified using a validated spectral unmixing algorithm as part of the instrument software (Aswendt et al., 2019; Gammon et al., 2006).

Herein, we introduce a novel click beetle mutant named CBG2. CBG2 paired with NH_2_-NpLH2 substrate (λ = 660 nm) can be integrated with the near-infrared system CBR2/NH_2_-NpLH2 (λ = 730 nm) (Hall et al., 2018) for dual-color near-infrared (NIR) BLI *in vivo*. We demonstrate that it is possible to spectrally resolve and quantify the bright emissions of CBG2 and CBR2 using a spectral unmixing algorithm. The high solubility and low toxicity associated with the salt form of NH_2_-NpLH2 luciferin make the system amenable to *in vivo* injection, thus expanding the BLI toolbox for measuring multiple biological processes in a single imaging session using a single luciferase substrate.

## Results

### Rational design of CBG2 luciferase and spectral characterization

Color variation in click beetle luciferases can be influenced at the protein level by a small number of amino acid positions. The best characterized mutants, CBG99, CBR, and CBR2, differ at only nine positions. To create a luciferase that can produce NIR emission suitable for multiplexing with CBR2 and efficiently utilize NH_2_-NpLH2, we chose CBG99 as our starting point. CBG99 was preferred over CBR primarily because of its narrower spectrum (Figure S1A). We first codon-optimized CBG99 luciferase (CBG99opt) to improve gene expression and protein levels in mammalian cells. CBG99opt has identical codons to CBR2 except at sites where there are amino acid differences. We confirmed that CBG99opt produces a spectral peak at 540 nm when combined with D-LH2 (Xu et al., 2016; Miloud et al., 2007) and a peak at 545 nm when used with NH_2_-NpLH2 (Table 1).

**Table 1:** Relative light unit (RLU) of CBR2, CBG99, and CBG2 measured with D-LH2 and NH_2_-NpLH2 in transiently transfected and lytic HEK293T cells

Purified Mutant Enzyme	D-LH2 lytic	Spectral Peak D-LH2 (nm)	NH_2_-NpLH2 lytic	Spectral Peak NH_2-_NpLH2 (nm)
CBR	0.96	620	26.72	660
CBR2	1.97	620	26.34	730
CBG99	0.83	540	0.58	545
CBG99opt	1.00	550	1.00	545
CBG2	4.38	585	14.72	660

Spectral peak data were acquired using purified enzymes.

**Figure 1:**
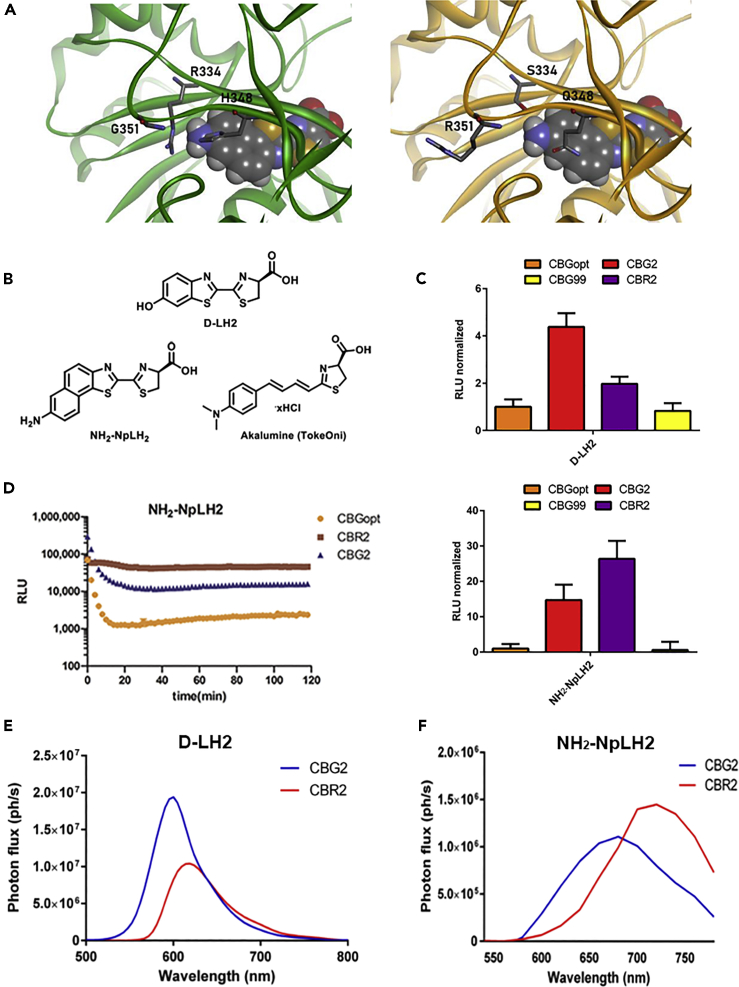
Rational design of CBG2 luciferase and spectral characterization For a Figure360 author presentation of this figure, see https://doi.org/10.1016/j.isci.2020.101986. (A) Homology models of CBG2 (left) and CBR2 (right) luciferases with bound NH_2_-NpLH2 substrate, based on firefly luciferase X-ray structure templates (PDB accession codes 2D1S, 4G36, and 5KYT). Residues that differ between CBG2 and CBR2 (334, 348, 351) are indicated. Modeling and rendering were performed using Discovery Studio software (BIOVIA). (B) Chemical structures of D-LH2, NH_2_-NpLH2, and AkaLumine-HCl substrates. (C) Bioluminescence intensity (RLU_max_) of CBG99, CBG99opt, CBR2, and CBG2 (cell lysates) combined with D-LH2 or NH_2_-NpLH2 substrates ( n= 3 samples; error bars represent ±SD). The spectra of CBG2/D-LH2 (top panel) and CBG2/NH_2_-NpLH2 (bottom panel) are presented as reference points. A summary of spectral peaks of the different combinations is reported in Table 1. (D) Kinetics of HEK293T CBG99opt, CBG2, and CBR2 cell lysets treated with NH_2_-NpLH2. Bioluminescence emission spectra for lysates containing CBG2 and CBR2 with D-LH2 (E) or NH_2_-NpLH2 (F). Spectra were acquired using an IVIS Spectrum with the following settings: FOV C, f/stop=1, medium binning, 30 s exposure time, and a range of band pass filters (500 nm to 780 nm).

Next, we designed a panel of mutants based on the nine amino acid differences between CBG99 and CBR2. The set included amino acid substitutions in the active site known to red-shift emission of beetle luciferases (Viviani et al., 2016). The mutant of highest interest that emerged from this analysis, CBG2, differs by six residues compared with CBG99 and by three residues compared with CBR2. CBG2 was red-shifted by 75 nm with NH_2_-NpLH2 (660 nm), when compared with the wild-type CBG99/NH_2_-NpLH2 (545 nm) (Table 1). A summary of the spectral characterization and brightness for the purified luciferase mutants is presented in Table 1. Residues that differ between CBR2 and CBG2 are highlighted in the structure model shown in Figure 1A. These residues are mainly located in the luciferin binding pocket of the enzymes and contribute to substrate affinity and color-shift (Woodroofe et al., 2008). We employed D-LH2 and its analogs NH_2_-NpLH2 and AkaLumine-HCl (depicted in Figure 1B) to evaluate the function of the novel mutant in this study.

Luminescence signals for CBR2 and CBG2 with D-LH2 and NH_2_-NpLH2 (100 μM) were measured in live cells and lysates (Figure 1C). With D-LH2 as substrate, CBG2 was ∼5-fold brighter and red-shifted ∼40 nm (to 585 nm) relative to both CBG99 and CBG99opt. Interestingly, the specificity of CBG2 in live cells is significantly higher than both CBG99 and CBG99opt with NH_2_-NpLH2, producing a 25-fold increase in light output with a ∼115 nm red-shift (660 nm). CBR2 luciferase yielded the brightest photon emission when used with NH_2_-NpLH2 and a near-infrared peak at 720 nm (Figure 1C). The CBR variant (also giving a peak at 660 nm) was considered for multiplex BLI, but further investigations in live cells revealed a broad, more intense spectral profile that significantly overlapped with the CBR2 spectrum (Figure S1A).

Kinetic properties for the mutant luciferases were tested in HEK293T live cells with 1.85 μM of NH_2_-NpLH2 (Figure 1D). After an initial loss (∼10-fold) in signal, CBG2 reached steady state after 10 min. The signal strength for CBR2 was higher compared with CBG2, but its signal duration was longer (Figure 1D). V_max_ and Km parameters of the enzymes with titrated D-LH2 or NH_2_-NpLH2 can be found in Figure S1B. Luminescence photon fluxes of HEK cells stably expressing CBG2, Luc2, CBR2, or Akaluc luciferases and their respective brightness with D-LH2, NH_2_-NpLH2, and Akalumine-HCl are highlighted in Figure S2A.

We attempted to unmix (i.e., resolve) the spectra of CBG2 and CBR2 with D-LH2 or NH_2_-NpLH2 using transfected HEK293T cells. When treated with D-LH2, CBG2 cells produced nearly 2-fold higher photon flux compared with CBR2. Emission peaks for the two systems were separated by 35 nm. This modest separation, combined with the broad emission spectrum for CBR2, prevented efficient resolution of signals (Figure 1E). In contrast, CBG2 cells treated with NH_2_-NpLH2 showed a consistent, red-shifted bioluminescent spectrum peaking at ∼660 nm and with a photon emission of 1.2 × 10^6^ ph s^−1^. This allowed enough spectral separation from the 730 nm peak for CBR2 (Figure 1F). Moreover, we confirmed sufficient spectral separation when CBR2 and CBG2 were co-transfected HEK cells. Use of the spectral unmixing tool allowed us to calculate the respective unmixed photon fluxes from cells expressing both luciferases (Figure S2B).

### *In vitro* kinetics and spectral unmixing of CBG2 and CBR2 luciferases

**Figure 2:**
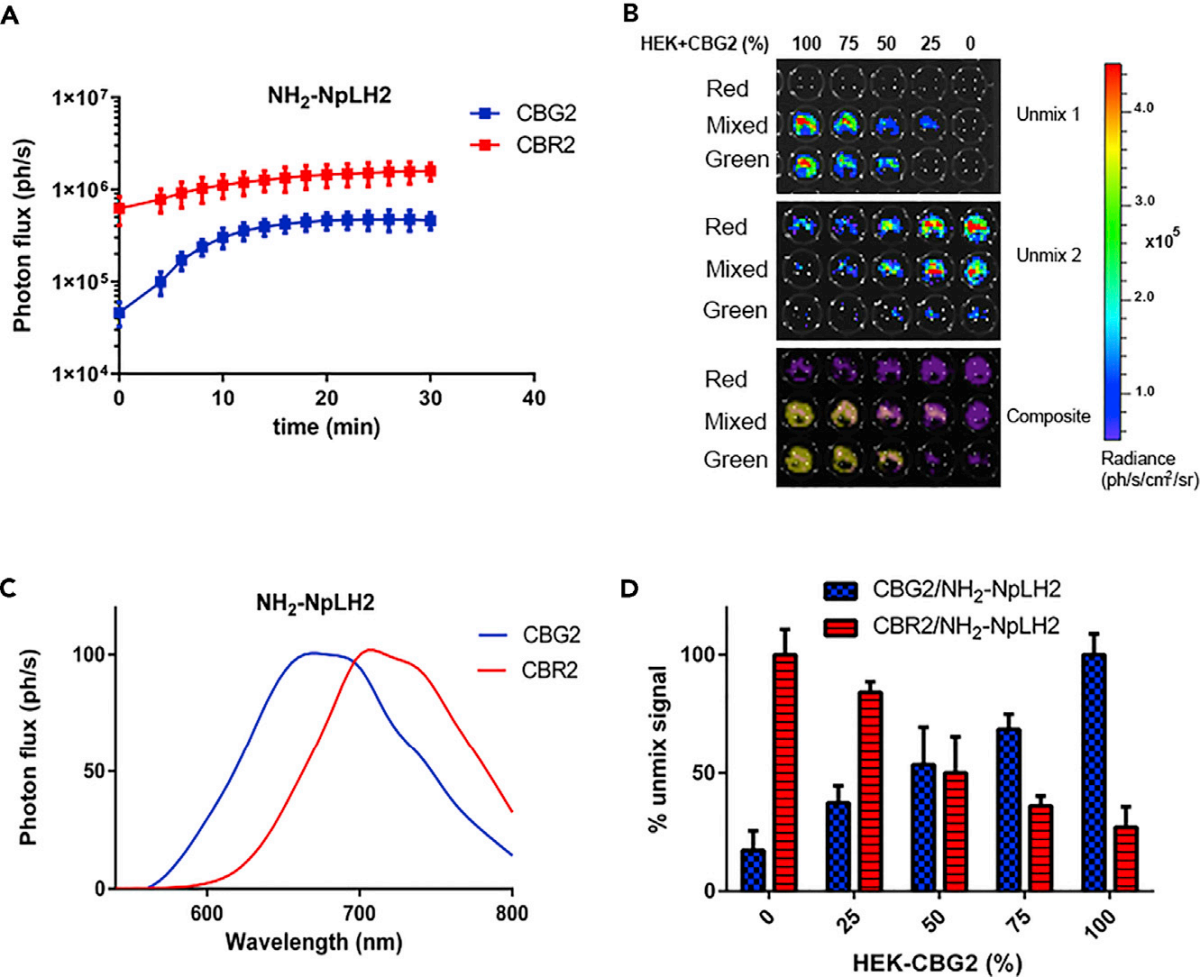
*In vitro* kinetics and spectral unmixing of CBG2 and CBR2 luciferases (A) Live-cell bioluminescence kinetics reported for HEK-CBG2 and HEK-CBR2 with NH_2_-NpLH2. Imaging was performed using an IVIS Spectrum with no filters and a 30 s exposure time. Imaging acquisitions were made every 2 min for a total of 15 acquisitions. This experiment was performed in triplicate; error bars represent ±SD. (B) Spectral unmixing of HEK cell expressing CBG2 or CBR2 and mixed in various proportions ranging from 100% to 0% of the total population. Plate was spectrally imaged using IVIS system. Spectral unmix was produced building a specific library for each pure luciferase/luciferin BLI and then applied to the mixture. (C) Normalized bioluminescence spectra generated by the spectral unmixing and revealing the feasibility to efficiently separate green and red spectra. Spectra were normalized to the peak emission for each Click beetle mutants with each substrate. (D) Quantification of the percentage unmixed signals of HEK-CBG2 and HEK-CBR2 with NH_2_-NpLH2. Unmixed signals were normalized to 100% cell ratios with p < 0.0001 and F-ratio 30.26 for mean values of HEK-CBG2 group and 31.82 for mean values of HEK-CBR2, calculated by one-way ANOVA. Error bars represent ±SD.

Kinetic profiles for CBG2 and CBR2 were measured in stable luciferase-expressing HEK cells *in vitro* (Figure 2A). To validate the spectral unmixing of HEK-CBG2 and HEK-CBR2 signals, cells expressing either CBG2 or CBR2 were plated in various ratios (ranging from 100% to 0%) in 96-well black plates (Figure 2B). Spectral imaging and unmixing were performed by selecting 14 band pass filters ranging from 540 to 800 nm on the IVIS spectrum using NH_2_-NpLH2 as substrate (1 mM). Interestingly, the algorithm was able to measure pure green signals (100% CBG2) and pure red signals (100% CBR2), making it possible to build a specific library for each luciferase contribution. The library was then applied to spectral unmixing. Figure 2C shows the successful unmixing of each spectrum, which then allowed us to plot the normalized and partially overlapped spectra of CBG2 (blue line) and CBR2 (red line). The same library was also used to quantify the photon flux of mixed green and red cell populations at different percentages between 100% and 0% (Figure 2D).

### *In vivo* characterization of CBG2 and CBR2 mutants and spectral unmixing

**Figure 3:**
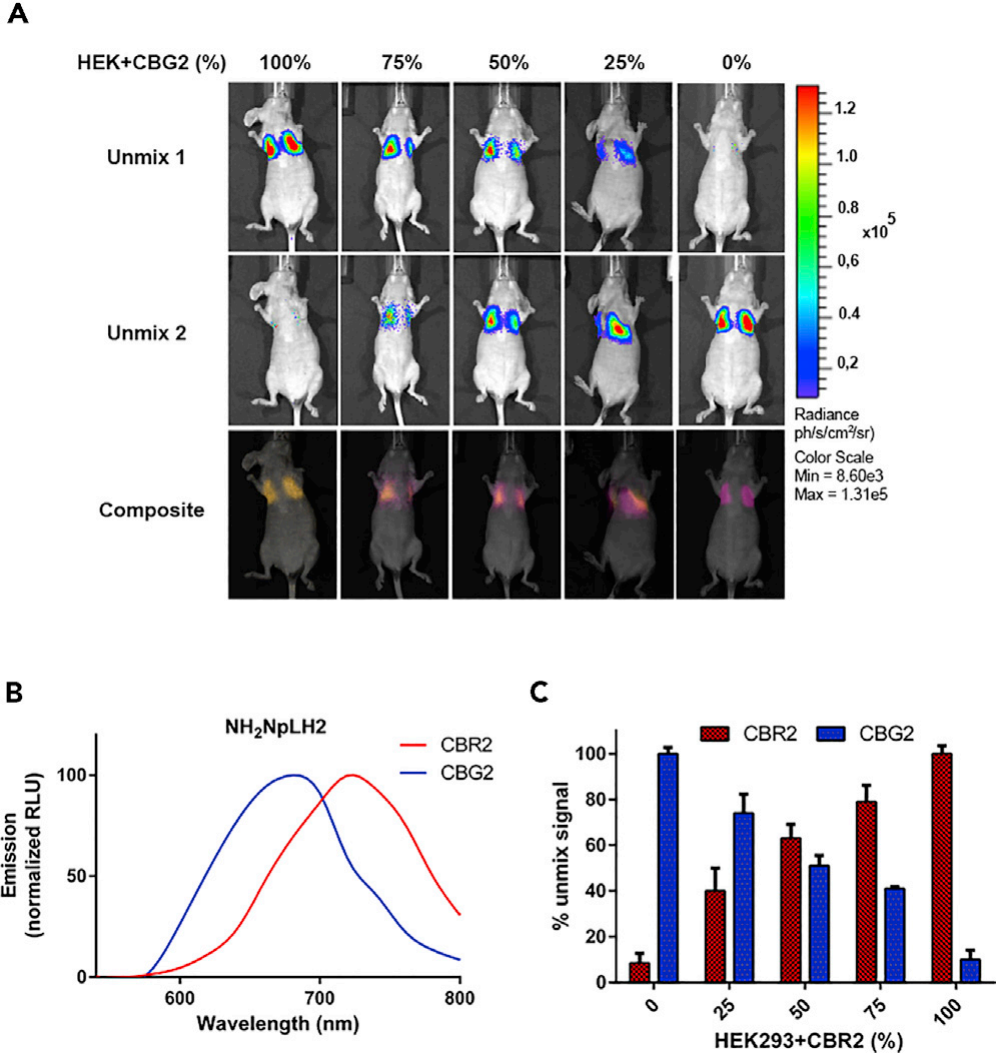
*In vivo* characterization of CBG2 and CBR2 mutants and spectral unmixing (A) Representative unmixed bioluminescence images of CBG2 and CBR2 with NH_2_-NpLH2 in deep tissue. Representative images of mice injected with different cell ratios of HEK-CBG2 and HEK-CBR2 with NH_2_-NpLH2 in a lung model (n = 3 samples). HEK-CBG2 or HEK-CBR2 cells were injected (I)v. at different proportions and NH_2_-NpLH2 substrate was injected (I)p. Images were acquired 15 min after substrate injection. Acquisition time for each filter was of 30 s. Band pass filters selected were between 540 nm and 800 nm. Filters selected for the green spectral unmixing is at 680 nm and for the red spectral unmixing is at 720 nm. Images were recorded 15 min after substrate injection considering the enzyme kinetics. Composite images indicate the linearity between the percentage of cells and the photons emitted. (B) Spectral proprieties HEK-CBG2 and HEK-CBR2 with NH_2_-NpLH2. (C) Quantification of the photon fluxes of the different percentages of HEK-CBG2/NH_2_-NpLH2 and HEK-CBR2/NH_2_-NpLH2 ranged from 100% to 0% as shown in Figure 3A. Bioluminescent unmixed signals were normalized to 100% cell ratios (n = 3 samples) with p < 0.05, F-ratio 4.064 for mean values of HEK-CBG2 group, and 16.33 for mean values of HEK-CBR2, calculated by one-way ANOVA. Error bars represent ±SD.

To validate the potentiality of the dual-color BLI system in deep tissue, we first injected HEK-CBG2 or HEK-CBR2 to build a guided library for pure green or red signals. Images were captured using an IVIS imager with 15 band pass filters ranging from 540 to 800 nm. Pure HEK-CBG2 and HEK-CBR2 or a mixture of the two cell types was injected following the schema: 100%‒0%; 75%‒25%; and 50%‒50% of green-red and then the same for red-green. NH_2_-NpLH2 substrate was injected intraperitoneally and photons flux was recorded 10 min after substrate injection. The spectral unmixing algorithm efficiently extracted green or red contributions at the different percentages (Figure 3A). Notably, unmixing was also successful when 25% of the total population of the unmixed green was injected. Quantitative analysis for the unmixed green and the unmixed red photon fluxes for each cell percentage (0%–25%; 50%–75%; 100%) was performed using Living Image software.. Figures 3B and 3C reveals a linear correlation between the percentage of cells injected and photons recorded for both HEK-CBG2 and HEK-CBR2.

### Versatility of CBG2 luciferase combined with AkaBLI system for dual color imaging

**Figure 4:**
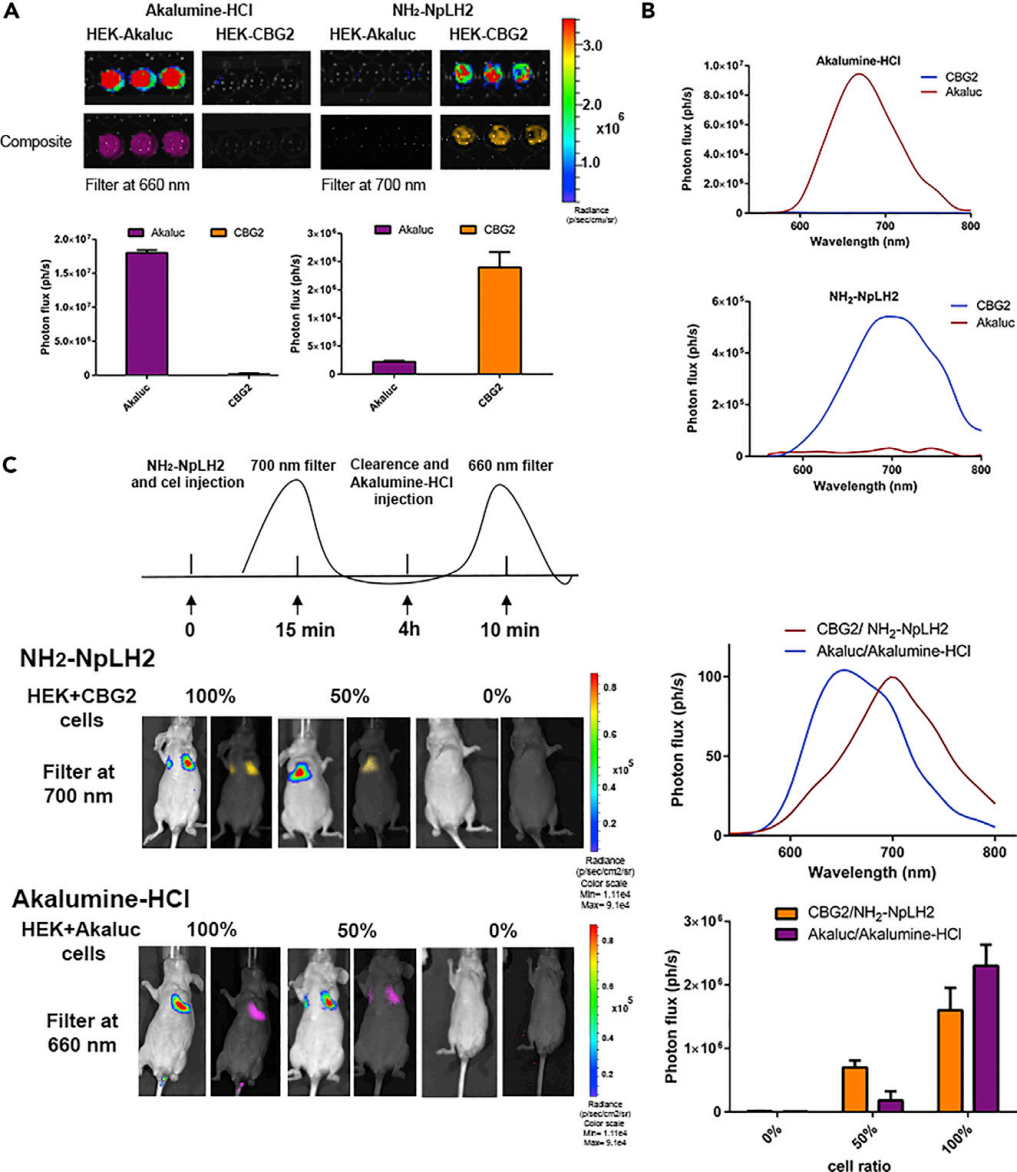
Versatility of CBG2 luciferase combined with AkaBLI system for dual color imaging (A) Representative live cell images of HEK-cells expressing Akaluc and CBG2 and tested with AkaLumine-HCl and NH_2_-NpLH2. Filters for the spectral unmixing were set at 660 nm for Akaluc/AkaLumine-HCl and at 700 nm for CBG2/NH_2_-NpLH2. Plots indicate photon fluxes for *in vivo* measurements. The experiment was performed in triplicate; error bars represent ±SD. (B) Spectral properties of HEK-Akaluc and HEK-CBG2 treated with AkaLumine-HCl and NH_2_-NpLH2 (0.1 mM). (C) Representative spectral unmixing *in vivo* (n = 3 samples) for 100%, 50%, or 0% of HEK-CBG2 or HEK-Akaluc with AkaLumine-HCl or NH_2_-NpLH2. Cells were injected intravenously and substrates were injected intraperitoneally. Images were acquired first 15 min after NH_2_-NpLH2 injection. Then, when pre-scan confirmed the clearance of NH_2_-NpLH2, AkaLumine-HCl was injected and images acquired 5 min after substrate injection. Guided libraries were generated to identify each signal by Living Image software (PerkinElmer). Normalized spectra generated (top-right panel) confirmed the feasibility to separate CBG2 and Akaluc signals *in vivo* by selecting filters at 660 nm for Akaluc/AkaLumine-HCl and 700 nm for CBG2/NH_2_-NpLH2. Acquisition time for each filter was 30 s. Quantification of the photon flux (bottom-right panel) at the different cell percentages (100%–50%–0%) for HEK-CBG2 or HEK-Akaluc with NH_2_-NpLH2 and for HEK-CBG2 or HEK-Akaluc with AkaLumine-HCl are plotted (right-bottom), p < 0.05, n = 3 samples; error bars represent ±SD.

We further explored whether CBG2/NH_2_-NpLH2 could be combined with Akaluc/AkaLumine-HCl (Iwano et al., 2018) for dual-color BLI. We selected two filters: 700 nm for CBG2/NH_2_-NpLH2 and 660 nm for Akaluc/AkaLumine-HCl. Akaluc yielded the brightest photon emission with AkaLumine-HCl (∼20-fold higher than CBG2/AkaLumine-HCl, Figure 4A). When the filter was set at 700 nm, CBG2/NH_2_-NpLH2 was ∼40-fold higher than Akaluc/NH_2_-NpLH2. Interestingly, the pairings CBG2/Akalumine-HCl and Akaluc/NH_2_-NpLH2 both recorded a dim signal, suggesting low enzyme activity for these combinations (Figure 4A). Thus, for dual-color BLI application the use of a single substrate where the enzymes have comparable expression was not feasible. Spectral curves and respective photon fluxes are depicted in Figure 4B. Next, we investigated whether a mixture of CBG2 or Akaluc cells could be measured using AkaLumine-HCl and NH_2_-NpLH2 for dual-color BLI. First, the original spectrum libraries were efficiently built with 100% CBG2 cells or 100% Akaluc cells using Living Image software (PerkinElmer). Each luciferase contribution was effectively separated and quantified Figure 4C (left). Separation could be achieved when the luciferase contributions were equal (50% HEK-CBG2 and 50% HEK-Akaluc). The spectral curves and quantification of luminescence signals at different cell ratios are depicted in Figure 4C (right).

## Discussion

We report here on a novel dual NIR click beetle luciferase system that can record semi-quantitative data from deep-tissue and whole-body imaging by reducing light attenuation caused by hemoglobin, melanin, and water. Importantly, the administration of NH_2_-NpLH2 as single substrate provides high sensitivity, reduces the number of animals required, and minimizes animal discomfort during the study (Cool et al., 2013). In comparison to previously published method (Stowe et al., 2019), the brightness of our system allowed for a substantial reduction in imaging time to 6 min or less (acquisition time per filter of 30 s instead of 120 s). Similar to previous reports using green luciferases, we observed some attenuation of CBG2 light emission due to the partial absorbance of the emitted green photons and a shift of the spectral peak to ∼680‒700 nm in deep tissue (Rumyantsev et al., 2016). However, the spectra of CBG2 and CBR2 maintained adequate spectral separation in the lungs, which allowed us to distinguish each luciferase contribution.

We also compared the versatility of the new CBG2/NH_2_-NpLH2 system with the recently developed AKA-BLI system (650 nm) (Iwano et al., 2018). This method can be exploited for multiplexed bioluminescence applications where Akaluc/AkaLumine-HCl and CBG2/NH_2_-NpLH2 can give distinct signals. Indeed, this setup will effectively probe more than one cellular process, each producing specific BL signals upon sequential administration of each substrate *in vivo*. However, this multiplex application will be less specific with CBR2/NH_2_-NpLH2 (730 nm) due to the bioluminescence recorded when AkaLumine-HCl is used (Hall et al., 2018; Zambito et al., 2020). Another limitation is that sequential substrate administration requires longer imaging sessions (Kleinovink et al., 2019; Yeh et al., 2019). Indeed, injection of two substrates requires the clearance of the first substrate but provides maximal light emission for each luciferase, thereby reducing spectral interference from each luciferase. Finally, we envision a highly sensitive triple color BLI application with CBG2/CBR2 with NH_2_-NpLH2 (680 nm and 720 nm, respectively) coupled with the novel optimized NanoLuc/hydrofurimazine (460 nm) in the same animal model (Su et al., 2020). This system (i.e., tri-plex BLI) could be used to visualize localization, activation, and other functional states of immune cells. In conclusion, a novel dual-color BLI in the NIR window can be accessed using CBG2 and CBR2 cells treated with a single substrate, NH_2_-NpLH2. This represents a promising approach for simultaneous visualization and quantification of two cell populations in deep tissue and in the same animal model. Collectively, this work will contribute to expand the toolset for *in vivo* multicolor bioluminescence imaging.

### Limitations of the study

The size and the photon flux of the signal area in deep tissue represent a potential limitation in the ability to efficiently separate and quantify the contribution of each luciferase. Although we demonstrated feasibility for lung imaging, we expect the method to be more accurate for dual color imaging of small areas (e.g., lymph nodes and depots of injected cells in deep organs) as demonstrated previously (Branchini et al., 2007). The kinetics of light emission after i.p. administration of NH_2_-NpLH2 is slow (BL signals peaking at ∼20 min), so repeated administration of substrate and imaging must be delayed by approximately 3 h. When CBG2/NH_2_-NpLH2 and Akaluc/AkaLumine-HCl are used for multiplex imaging, sequential administration of the two substrates is needed. This may require longer imaging sessions depending on substrate properties as solubility, enzyme affinity, different bio-distribution, and serum stability (Yeh et al., 2019).

### Resource availability

#### Lead contact

Further information and requests should be directed to the Lead Contact, Dr. Laura Mezzanotte (l.mezzanotte@erasmusmc.nl)

#### Materials availability

Materials are available from the corresponding author on request.

#### Data and code availability

This study did not generate computer code. All data and analytical methods are available in the main text or in Supplemental information.

## Methods

All methods can be found in the accompanying Transparent Methods supplemental file.

## Supplemental information

Document S1. Transparent methods and Figures S1 and S2

Figure360. An Author Presentation of Figure 1

## Footnotes

Supplemental information can be found online at https://doi.org/10.1016/j.isci.2020.101986.

## References

[bib1] Aswendt M., Voge l. S., Schäfer C., Jathoul A., Pule M., Hoehn M. (2019). Quantitative in vivo dual-color bioluminescence imaging in the mouse brain. Neurophotonics.

[bib2] Branchini B.R., Ablamsky D.M., Murtiashaw M.H., Uzasci L., Fraga H., Southworth T.L. (2007). Thermostable red and green light-producing firefly luciferase mutants for bioluminescent reporter applications. Anal. Biochem..

[bib3] Branchini B.R., Southworth T.L., Fontaine D.M., Kohrt D., Florentine C.M., Grossel M.J. (2010). Red-emitting luciferases for bioluminescence reporter and imaging applications. Anal. Biochem..

[bib4] Cool S.K., Breyne K., Meyer E., De Smedt S.C., Sanders N.N. (2013). Comparison of in vivo optical systems for bioluminescence and Fluorescence imaging. J. Fluoresc..

[bib5] Gammon S.T., Leevy W.M., Gross S., Gokel G.W., Piwnica-Worms D. (2006). Spectral unmixing of multicolored bioluminescence emitted from heterogeneous biological sources. Anal. Chem..

[bib6] Hall M.P., Woodroofe C.C., Wood M.G., Que I., van’t Root M., Ridwan Y., Shi C., Kirkland T.A., Encell L.P., Wood K.V. (2018). Click beetle luciferase mutant and near infrared naphthyl-luciferins for improved bioluminescence imaging. Nat. Commun..

[bib7] Iwano S., Sugiyama M., Hama H., Watakabe A., Hasegawa N., Kuchimaru T., Tanaka K.Z., Takahashi M., Ishida Y., Hata J. (2018). A Single-cell bioluminescence imaging of deep tissue in freely moving animals. Science.

[bib8] Jathoul A.P., Grounds H., Anderson J.C., Pule M.A. (2014). A dual-color far-red to near-infrared firefly luciferin analogue designed for multiparametric bioluminescence imaging. Angew. Chem. Int. Ed..

[bib9] Kaskova Z.M., Tsarkova A.S., Yampolsky I.V. (2016). 1001 lights: luciferins, luciferases, their mechanisms of and applications in chemical analysis, biology and medicine. Chem. Soc. Rev..

[bib10] Kleinovink J.W., Mezzanotte L., Zambito G., Fransen M.F., Cruz L.J., Verbeek J.S., Chan A., Ossendorp F., Löwik C. (2019). A dual-color bioluminescence reporter mouse for simultaneous in vivo imaging of T cell localization and function. Front. Immunol..

[bib11] Maguire C.A., Bovenberg M.S., Crommentuijn M.H., Niers J.M., Kerami M., Teng J., Sena-Esteves M., Badr C.E., Tannous B.A. (2013). Triple bioluminescence imaging for in vivo monitoring of cellular processes. Mol. Ther. Nucleic Acids.

[bib12] Mezzanotte L., Que I., Kaijzel E., Branchini B., Roda A., Löwik C. (2011). Sensitive dual color in vivo bioluminescence imaging using a new red codon optimized firefly luciferase and a green click beetle luciferase. PLoS One.

[bib13] Mezzanotte L., van ‘t Root M., Karatas H., Goun E.A., Löwik C. (2017). In vivo molecular bioluminescence imaging: new tools and applications. Trends Biotechnol..

[bib14] Miloud T., Henrich C., Hammerling G.J. (2007). Quantitative comparison of click beetle and firefly luciferases for in vivo bioluminescence imaging. J. Biomed. Opt..

[bib15] Rumyantsev K.A., Turoverov K.K., Verkhusha V.V. (2016). Near-infrared bioluminescent proteins for two-color multimodal imaging. Sci. Rep..

[bib16] Smith A.M., Mancini M.C., Nie S. (2009). Bioimaging: second window for in vivo imaging. Nat. Nanotechnol..

[bib17] Stowe C.L., Burley T.A., Allan H., Vinci M., Kramer-Marek G., Ciobota D.M., Parkinson G.N., Southworth T.L., Agliardi G., Hotblack A. (2019). Near-infrared dual bioluminescence imaging in mouse models of cancer using infraluciferin. Elife.

[bib18] Su Y., Walker J.R., Park Y., Smith T.P., Liu L.X., Hall M.P., Labanieh L., Hurst R., Wang D.C., Encell L.P. (2020). Novel NanoLuc substrates enable bright two-population bioluminescence imaging in animals. Nat. Methods.

[bib19] Taylor A., Sharkey J., Plagge A., Wilm B., Murray P. (2018). Multicolour in vivo bioluminescence imaging using a nanoluc-based bret reporter in combination with firefly luciferase. Contrast Media Mol. Imaging.

[bib20] Viviani V.R., Simões A., Bevilaqua V.R., Gabriel G.V.M., Arnoldi F.G.C., Hirano T. (2016). Glu311 and Arg337 stabilize a closed active-site conformation and provide a critical catalytic base and countercation for green bioluminescence in beetle luciferases. Biochemistry.

[bib21] Wilson T., Hastings J.W. (1998). Bioluminescence. Annu. Rev. Cell Dev. Biol..

[bib22] Woodroofe C.C., Shultz J.W., Wood M.G., Osterman J., Cali J.J., Daily W.J., Meisenheimer P.L., Klaubert D.H. (2008). N-Alkylated 6′-aminoluciferins are bioluminescent substrates for Ultra-Glo and QuantiLum luciferase: new potential scaffolds for bioluminescent assays. Biochemistry.

[bib23] Xu T., Close D., Handagama W., Marr E., Sayler G., Ripp S. (2016). The expanding toolbox of in vivo bioluminescent imaging. Front. Oncol..

[bib24] Yeh H.-W., Wu T., Chen M., Ai H.-W. (2019). Identification of Factors complicating bioluminescence imaging. Biochemistry.

[bib25] Zambito G., Natasa G., Ridwan Y., Hall M.P., Shi C., Kirkland T.A., Encell L.P., Löwik C., Mezzanotte L. (2020). Evaluating brightness and spectral properties of click beetle and firefly luciferases using luciferin analogues: identification of preferred pairings of luciferase and substrate for in vivo bioluminescence imaging. Mol. Imaging Biol..

