# Supplementary material for: Red-shifted click beetle luciferase mutant expands the multicolor bioluminescent palette for deep tissue imaging
Source: iScience. 2020 Dec 26;24(1):101986. doi: 10.1016/j.isci.2020.101986 (PMC7811125; doi:10.1016/j.isci.2020.101986)
Supplement: Document S1. Transparent methods and Figures S1 and S2 [file mmc1.pdf]

iScience, Volume *24*

Supplemental Information

Red-shifted click beetle luciferase mutant expands the multicolor bioluminescent palette for deep tissue imaging

Giorgia Zambito, Mary P. Hall, Monika G. Wood, Natasa Gaspar, Yanto Ridwan, Fabio F. Stellari, Ce Shi, Thomas A. Kirkland, Lance P. Encell, Clemens Löwik, and Laura Mezzanotte

Supplemental Information

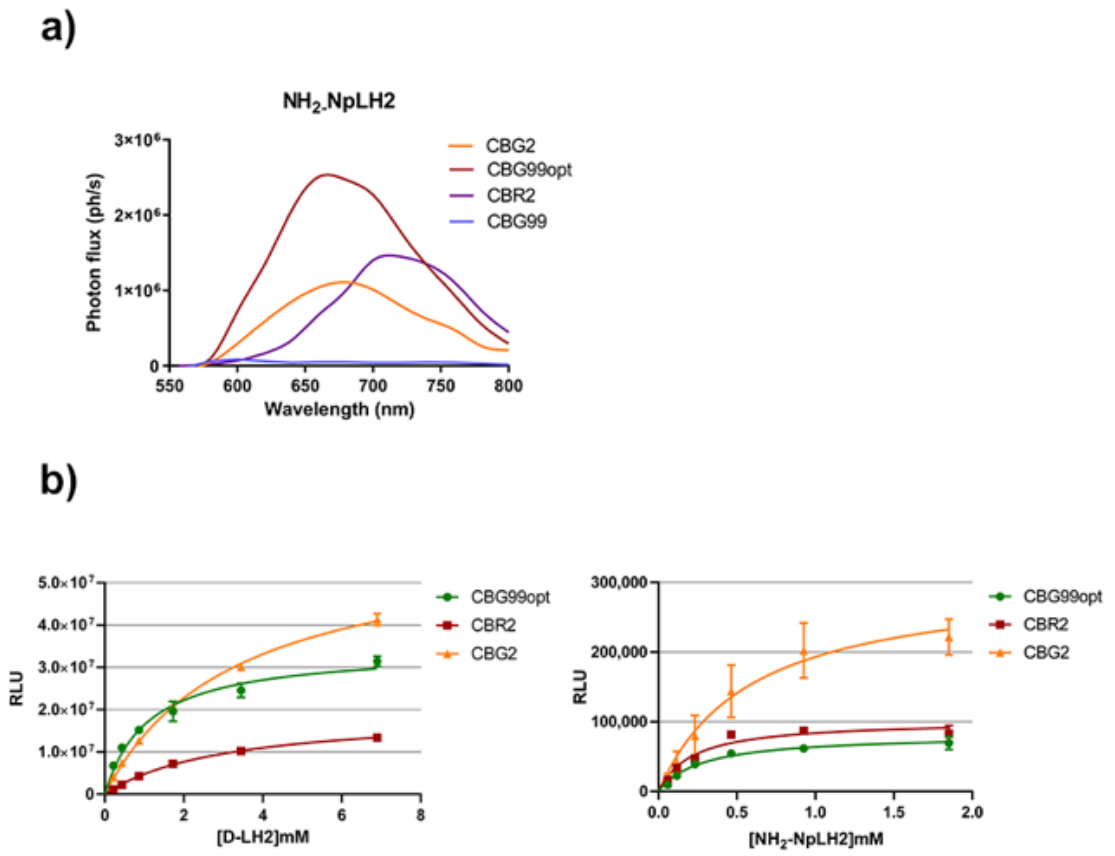

	CBG99opt	CBR2	CBG2
Vmax	3.441e+007	1.933e+007	6.061e+007
Km	1.096	3.044	3.318

	CBG99opt	CBR2	CBG2
Vmax	81510	101820	305577
Km	0.2758	0.2150	0.5812

Figure S1. In vitro characterization of click beetle luciferase variants, Related to Figure 1. (a) Live HEK293T cell lines were transfected with plasmids expressing CBR2, CBG2, CBG99 or CBG99opt luciferase genes. Photon fluxes were quantified after treatment with NH_2-NpLH2 substrate (1mM) by the IVIS imager. Spectral curves were built by Living image software 4.5 (Perkin Elmer). **(b)** V_{max} and Km parameters of the CBG99opt, CBG2 and CBR2 enzymes with titrated D-LH2 or NH_2-NpLH2. Km and RLU_{max} values were calculated using GraphPad Prism (Michaelis–Menten regression).

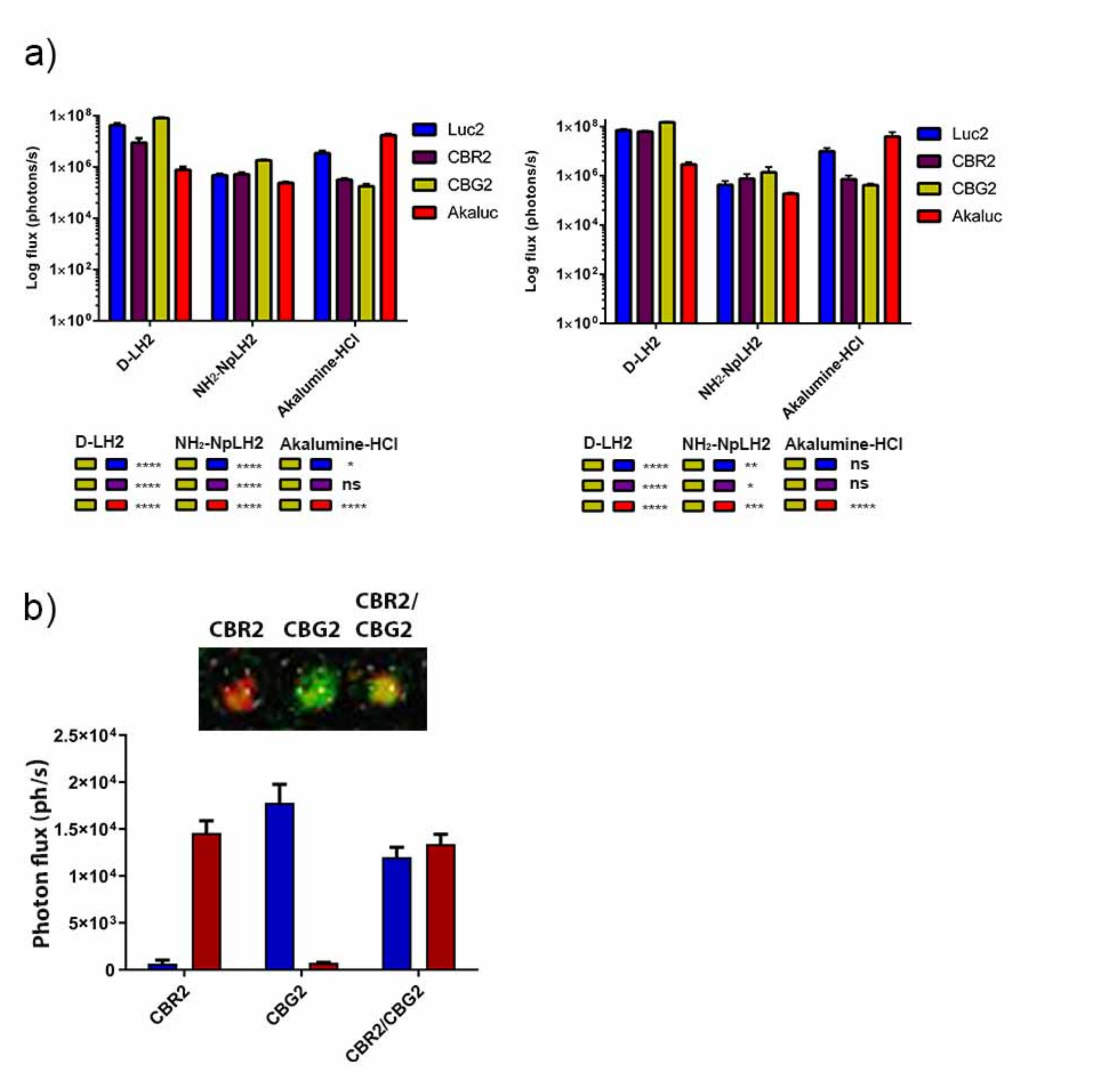

Figure S2. In vitro measurements of the photon fluxes and two-population luciferase brightness test. Related to Figure 2. (a) Live HEK293T cells stably expressing Luc2, CBR2, CBG2 and Akaluc luciferases, were tested for their brightness after addition of 0.1 mM (left panel) and 1 mM (right panel) of D-LH2 or analogues. Quantifications were performed 10 min after substrate addition. BL signals were compared to the brightness of CBG2 and statistical analysis was performed by One-way ANOVA, $p<000.1$. Experiment was performed in triplicate (n=3 samples). Error bars represent ±SD. **b)** In vitro two-population dual-color imaging of transfected HEK293T cells expressing CBR2 or/and CBG2 and respective quantification of the unmixed photon fluxes by spectral unmixing algorithm (n=3 samples).

Transparent Methods

Click beetle mutant construction. CBG99 and CBR wild type were codon optimized to match the codon usage in CBR2. Substitutions known to red shift CBG99 were added to the codon optimized CBG99 template, while substitutions known to blue shift CBR were added to the codon optimized CBR template. Substitutions of interest were combined on a common template to arrive at CBG2. Plasmids were constructed by Gene Dynamics, LLC.

Screening of luciferase mutants in live and lytic cell assay

Media was aspirated from a confluent flask of HEK293 cells (ATCC Hek293 cell (CRL1573). Cells were washed with DPBS (10 ml) (Life Technologies 14190) and then detached from the flask with TrypLE Express Trypsin (3 ml) (Life Technologies (12604). Cells were counted and resuspended to a concentration of 100,000 cell ml^{-1} in DMEM media supplemented with FBS (10%) and then diluted cells (100 µl) of was added to each well of a 96 well assay plate. Plates were grown for 24 h at 37 °C with 5% CO_2. The following day transfection complexes were prepared for each construct (CBGopt, CBR2, CBG2) which consisted of DNA (1 µg) diluted in a final volume of OptiMEM (50 µl) (11058) with Fugene transfection reagent (3.3 µl) (Promega E2311). Transfection complexes were incubated (15 min) and then each complex (5 µl) was added to wells of assay plates for each sample. Samples were then incubated for an additional (24 h). The following day both lytic and live cell assays were performed on each sample. For the live cell readings substrates were titrated as described below for Km and RLU_{max} determination. For the lytic reading BrightGlo assay buffer (Promega E264B) supplemented with ATP (1 mM) and D-Luciferin (100 µM) or NH_2-NpLH2 (100 µM) was added to 4 wells for each sample. Plates were placed on an orbital shaker at 600 RPM for 3 or 10 min and then luminescence was measured on a GMM+ GloMax® Multi+ for D-Luciferin or LAS 4000 CCD imager for NH_2-NpLH2.

Enzyme characterization: Km and RLU_{max} determination

The following substrate solutions were prepared in DPBS: D-LH2 (30 mM) (Promega), NH_2-NpLH2 (8 mM). Two-fold serial dilutions were prepared for each substrate (30 mM to 0.93 mM for D-LH2 or 8 mM to 0.25 mM for NH_2-NpLH2). Prior to assay, media from transfected wells was aspirated and replaced with CO_2 independent media supplemented with FBS (10%). Substrate dilutions (30 µl) were added to cells transfected with either CBGopt, CBG2 and CBR2. The plate was manually shaken, and immediately placed in a GloMax®-Multi+ luminometer (Promega) set to 37 °C. Kinetic reads were obtained for each substrate and sample combination. Initial timepoints were used to determine Km and RLU_{max} for each substrate. Km and RLU_{max} values were calculated using GraphPad Prism (Michaelis–Menten regression).

Cell culture and Preparation for cell assays

HEK293T cells were grown in DMEM medium (Sigma, St. Louis, Mo, USA) supplemented with FBS (10%) and of penicillin (1%) and streptomycin (1%) and incubated at 37 °C with 5% CO_2. When cell confluence reached around 80%, cells were washed with DPBS (life technologies 14190) and detached with 1 ml of Trypsin. Cells were centrifuged and re-suspended with fresh new medium (8 ml). Cell counting was performed using BioRad TC20 cell counter.

Cell transfection

HEK293T were plated at the density of 2 x 10^4 cells per well in a black 96 well plate (100 µl of complete DMEM). DNA plasmid of each luciferases (LUC2, CBG2, CBR2 or CBRopt) was diluted (at the concentration of 0.020 µg $µl^{-1}$ in 16 µl of sterile deionized water) to transfect 3 wells. Fugene® HD reagent (1 µl) was added to the complex and after mixing carefully, it was incubated for 10 min at room temperature. the preparation (5 µl) was added to each well of previously plated HEK293T. Plate was incubated at 37 ºC with 5% of CO_2 for 24 h.

Lentiviral production

Virus production and cell transduction were performed under appropriate biosafety level conditions (ML-II) in accordance with the National Biosafety Guidelines and Regulations for Research on Genetically Modified Organisms. Procedures and protocols were reviewed and approved by the EMC Biosafety Committee (GMO permit 99-163). The lentiviral plasmids pCDH-EF1-CBG2-T2A-copGFP, pCDH-EF1-CBR2-T2A-copGFP were used to transfect HEK-293 T cells with three packaging plasmids (pCMV-VSVG, pMDLg-RRE, pRSV-REV; Addgene, Cambridge, MA, USA) and the lentiviral vector plasmids using PEI transfection reagent (1mg/ml)/µg DNA) as previously described (Mezzanotte et al. 2011). The supernatant containing lentiviral particles were collected 48 h and 72 h after infection. Subsequent quantification of the virus was performed using a standard antigen-capture HIV p24 ELISA (ZeptoMetrix Corporation, NY, USA). Lentiviral plasmid of pCDH-EF1-Akaluc-T2A-copGFP was produced as previously described (Zambito et al. 2020).

Cell transduction

Cell transduction was performed by culturing HEK293T cells in complete DMEM at the density of 200,000 cells in a T25-flask with medium (5 ml). Expression in the lentiviral plasmid is driven by housekeeping elongation factor 1α (EF1) promoter. Cells were transduced with MOI 1 of either pCDH-EF1-CBG2-T2A-copGFP, pCDH-EF1-CBR2-T2A-copGFP with Polybrene (hexametride bromide, Sigma-Aldrich) at the final concentration (8 µg ml^{-1}). Cell were then expanded and sorted two times for copGFP (copepod Potenilla plumata) expression by FACS (BD-FACS AriaIII, BD Biosciences). HEK293T cells were transduced with pCDH-EF1-Akaluc-T2A-copGFP as previously described (Zambito et al. 2020).

***In vitro* bioluminescence imaging**

For in vitro BL imaging, HEK293T cells were plated at a density of 1 x 10^4 cells per well in a black 96-well plate (Greiner Cell Star®). Cells were transfected with DNA plasmids of CBG2, CBR2 or

simultaneously transfected with both plasmids. Prior imaging session, cells were washed and resuspended in PBS.

For the other tests with HEK-EF1-CBG2-T2A-copGFP, HEK-EF1-CBR2-T2A-copGFP and HEK-EF1-Akaluc-T2A-copGFP, cells were plated in triplicate in a black 96-well plate at the density of 1 x 10^4 cells per well and incubated at 37 °C for 24 h. Prior imaging session, cells were washed and resuspended in PBS. For kinetic study of HEK-EF1-CBG2-T2A-copGFP and HEK-EF1-CBR2-T2A-copGFP, imaging was performed after addition of NH_2-NpLH2 (final concentration 0.1 mM). Imaging settings were made at the IVIS spectrum system with open filter, 30 s exposure time, FOV C, f/stop=1, medium binning. Imaging acquisitions were made every 2 min for a total of 15 acquisitions. This experiment was performed in triplicate. For all other in vitro measurements, imaging was performed after addition of D-LH2, NH_2-NpLH2 or Akalumine-HCl (final concentration of 1 mM resuspended in PBS; 100 µl per well of a 96 black well plate). To accurately measure photon output while taking the kinetic profiles into account, images were acquired 10 min after addition of D-LH2 or NH_2-NpLH2 substrates and 5 min after addition of the Akalumine-HCl substrate. Images were acquired with the following settings: FOV C, f/stop=1, medium binning, 30 s exposure time and open filter. Images were acquired selecting a series of band pass filters ranged from 540 nm to 800 nm on the IVIS Spectrum (20 nm bandpass from 560 nm to 800 nm). ROI analysis of the spectral profiles and spectral unmixing were made on Living image software 4.5 (Perkin Elmer).

For spectral unmixing analysis, guided spectral unmixing of pure (100%) HEK-CBG2 cells, HEK-CBR2 or HEK-Akaluc cells was created. This allowed us to obtain a specific library of spectra for each luciferase and a distinct spectral signature for each luciferase/substrate. The specific library of spectra was then used to perform the spectral unmixing on a mixture of cell populations. Therefore, the relevant library of spectra allowed us to quantify the photons derived from the mixed cell population and to distinguish contributions from HEK-CBG2, HEK CBR2 or HEK-Akaluc. The

spectral unmixing allowed also to draw respective spectral profiles for HEK-CBG2, HEK-CBR2 and HEK-Akaluc. Experiments were repeated three times with three replicates.

In vivo animal model

Animal studies were approved by the Ethical Committee of Erasmus Medical Center, Rotterdam, The Netherlands. Animal care and handling was in accordance with the guidelines and regulations as stipulated by the Dutch Experiments on animal act (WoD) and The European Directive in the protection on animal used for scientific purposes (2010/63/EU). BALB/C nude (females) were purchased from Charles River Laboratory (The Netherlands). All mice aged 6–10 weeks were provided access to food and water *ad libitum* and were hosted in the animal facility at the Erasmus MC, Rotterdam, The Netherlands.

In vivo bioluminescence imaging: dual-color imaging, spectral unmixing and sequential imaging

Animal experiments were performed with mice anesthetized using isoflurane (1.5%). Groups of 3 mice were used for each condition. For the deep tissue model, HEK-EF1-CBG2-T2A-copGFP and HEK-EF1-CBR2-T2A-copGFP, HEK-EF1-Akaluc-T2A-copGFP were injected intravenously (i.v.) at a density of 1 million cells in 100 µl DPBS. NH_2-NpLH2 (220 mg Kg^{-1}) or Akalumine-HCl (50 mg Kg^{-1}) substrates were injected intraperitoneally (i.p.) (200 µl). In the case where animals received 50/50, 75/25 or 25/75 ratio of HEK-EF1-CBG2-T2A-copGFP and HEK-EF1-CBR2-T2A-copGFP, $5x10^5/5x10^5$, $7.5x10^5/2.5x10^5$ and $2.5x10^5/7.5x10^5$ cells were i.v. injected.

The dose of NH_2-NpLH2 or Akalumine-HCl substrates injected intraperitoneally were calculated based on the maximum solubility (especially for Akalumine-HCl), tolerability in mice and maximum attainable signal on previous findings (Branchini et al. 2010; Gammon et al. 2006). Images were acquired by IVIS spectrum (FOV C, binning medium, f/stop=1, 20 nm). Band pass filters were ranged between 540 nm to 800 nm and exposure time of 30 s was optimal for quantification of

bioluminescent signals required by spectral unmixing.). The series of images acquired with filters were taken 10, 16, and 21 min after i.p. injection of the substrates to determine the timing for maximal signal output. The stage was heated to 37 ºC. Open filters were used prior to the spectral recordings to assess stability of the substrate.

Spectral analysis and spectral unmixing of the in vivo images were performed by drawing ROIs with Living image software (Perkin Elmer). For guided spectral unmixing pure CBG2, CBR2 and Akaluc bioluminescent signals were recorded when combined with appropriate substrate. Once pure libraries were built, the relevant library spectra were then used to distinguish each luciferase contributions by spectral unmixing in the lung model. The spectral properties for each luciferase were drawn and quantified using the spectral unmixing algorithm.

For sequential imaging of HEK-CBG2 and HEK-Akaluc, mice were first injected with HEK-CBG2 cells ($1x10^6$) i.v. To quantify the bioluminescent signal required by spectral unmixing, the imaging session started 15 min after i.p. injection of NH2-NpLH2 (220 mg Kg^{-1}) using band pass filters that ranged between 540 nm to 800 nm with an exposure time of 30 s. Once the imaging session for HEK-CBG2/ NH2-NpLH2 was completed, clearance of the substrate was monitored after approximately 4 h by performing a pre-scan to access the absence of the signal. Then, HEK-Akaluc cells were injected i.v. ($1x10^6$) and Akalumine-HCl i.p. (50 mg Kg^{-1}). Images were acquired 5 min after substrate addition using band pass filters that ranged between 540 nm to 800 nm with exposure time of 30 s. Bioluminescent signals were quantified by spectral unmixing. All mice were culled at the end of the in vivo imaging sessions.

Statistical analysis

Km and RLU_{max} values were calculated using GraphPad Prism (Michaelis–Menten regression). The other in vitro and in vivo tests were performed using Graphpad 7 software and T test and ONE-way ANOVA. Results reported as mean ±SD and significance attributed when $p< 0.001$ (*) for in vitro tests or $p< 0.05$ (*) for in vivo tests.

Supplemental References

Mezzanotte, L., Que, I., Kaijzel, E., Branchini, B., Roda, A., Löwik, C. (2011) Sensitive dual color in vivo bioluminescence imaging using a new red codon optimized firefly luciferase and a green click beetle luciferase. PLoS One 6(4).

Zambito, G., Natasa, G., Ridwan, Y., Hall M.P., Shi, C., Kirkland, T.A., Encell, L. P., Löwik, C., Mezzanotte, L. (2020). Evaluating brightness and spectral properties of click beetle and firefly luciferases using luciferin analogues: identification of preferred pairings of luciferase and substrate for in vivo bioluminescence imaging. Molecular Imaging and Biology volume 22, 1523–1531.

Branchini, B.R., Southworth, T.L., Fontaine, D.M., Kohrt, D., Florentine, C.M., Grossel, M.J. (2010). Red-emitting luciferases for bioluminescence reporter and imaging applications. Anal Biochem. 396(2):290-297.

Gammon, S. T., Leevy, W. M., Gross, S., Gokel, G. W. & Piwnica-Worms, D. (2006). Spectral unmixing of multicolored bioluminescence emitted from heterogeneous biological sources. *Anal. Chem.* 78, 1520–1527.
